# Sporadic haemangioblastoma of the kidney with rhabdoid features and focal CD10 expression: report of a case and literature review

**DOI:** 10.1186/1746-1596-7-39

**Published:** 2012-04-12

**Authors:** Wei-hua Yin, Jian Li, John KC Chan

**Affiliations:** 1 Department of Pathology, Peking University Shenzhen Hospital, No.1120 Lianhua North Road, Shenzhen 518000, China; 2 Department of Pathology, Queen Elizabeth Hospital, Hong Kong, SAR, China

**Keywords:** Haemangioblastoma, Kidney, CD10, Rhabdoid features

## Abstract

We present here an intriguing case of sporadic renal haemangioblastoma occurring in a 61-year-old male. The tumor consisted of nests of polygonal cells and abundant capillary networks. The neoplastic cells generally showed abundant eosinophilic cytoplasm and prominent eccentric nuclei, resembling the rhabdoid cells. Pronounced intranuclear cytoplasmic pseudoinclusions were another significant feature seen. NSE, a-inhibin and S100 were positive in tumor cells and particularly, focal CD10 expressions were observed. This is possibly the first reported case of a haemangioblastoma showing a rhabdoid phenotype and CD10 immunopositivity. Malignant rhabdoid tumor and renal cell carcinoma with rhabdoid features were probably the most challenging mimics need to be differentiated. The result of focal CD10 staining in our case may further lead to confusion with renal cell carcinoma. To avoid misdiagnosis, more considerations should be attached to the rare neoplasm.

**Virtual Slides:**

The virtual slide(s) for this article can be found here: http://www.diagnosticpathology.diagnomx.eu/vs/1068858553657049

## Background

Haemangioblastoma is a slowly growing, highly vascular benign tumor, corresponding to WHO grade I. It typically arises within the central nervous system (CNS), but may occasionally originate in unusual sites such as peripheral nerve, bone, soft tissue, skin, liver, lung and pancreas [1-3], and maybe associated with von Hippel-Lindau (VHL) disease.

The kidney is another rare site for the development of sporadic haemangioblastoma growth, and only four cases have been reported in the English-language literature so far [4-6]. The accurate diagnosis is often challenging when the tumor develops in this region. We described herein the fifth case of this rare tumor, which notably demonstrated a rhabdoid phenotype as well as unexpected CD10 staining. In addition, the shared characteristics of renal haemangioblastomas (RHB) and their differential considerations were also discussed in detail.

### Case presentation

A 61-year-old man was admitted to our hospital for a solid mass found in the right kidney during a routine checkup. Computed tomography showed that the mass was located in the superior pole. No remarkable symptoms such as flank pain or urinary irritation were reported by the patient. He also had no familial history or clinical evidences of VHL disease. Radical nephrectomy was carried out, showing a 5.3 × 5.0 × 5.0 cm mass. It was grey to yellowish in color and well-demarcated from the surrounding renal parenchyma. The patient had an uneventful postoperative recovery and was well at 12 months follow-up.

### Histopathological and Immunohistochemical findings

**Figure 1:**
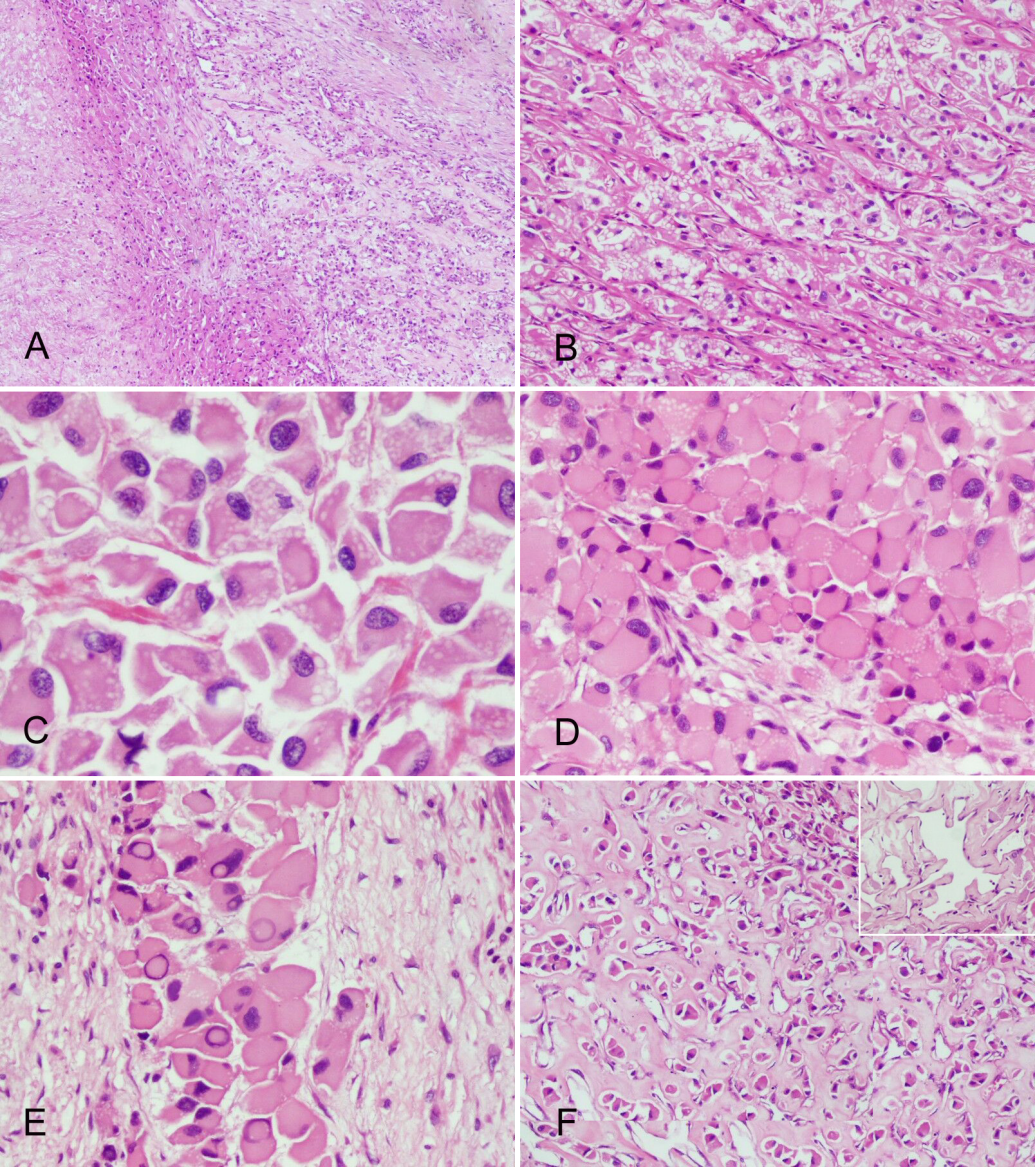
**Histopathological findings of the renal hemangioblastoma**. (**A**) Stromal hyalinization was prominent among the neoplasm and foci of necrosis were observed inside the tumor (left field) (H&E staining, with original magnification ×40). (**B**) The tumor cells were arranged in nests and traversed by a vascular network (H&E staining, with original magnification ×100). (**C**) Lipid vesicles were abundant in some tumor cells (H&E staining, with original magnification ×400). (**D**) The tumor cells had enlarged eosinophilic cytoplasm and eccentrically-displaced nuclei, exhibiting a rhabdoid phenotype (H&E staining, with original magnification ×200). (**E**) Pseudo-nuclear invaginations were another distinctive feature of tumor cells (H&E staining, with original magnification ×400). (**F**) The sclerotic stroma dispersed tumor cells into isolated small nests (H&E staining, with original magnification ×100). The vessels in between were usually dilated and some of them resembled the changes of papillary endothelial hyperplasia (inserted panel, H&E staining, with original magnification ×400).

Microscopically, the specimen consisted of nests of polygonal tumor cells and a prominent capillary network. Focal areas of necrosis were present. The stroma showed extensive fibrosis and hyalinization, which amounted to approximately one third of the lesion (Figure 1A, B).

The majority of the neoplastic cells were enlarged with marked eosinophilic cytoplasm that sometimes contained sharply delineated lipid vacuoles. A few cells showed highly vacuolated and clear cytoplasm. The nuclei were generally eccentric, mildly to moderately pleomorphic with coarse granular chromatin, resembling the rhabdoid cells (Figure 1C, D). The nucleoli were inconspicuous. However, there were many prominent intranuclear cytoplasmic pseudoinclusions (Figure 1E). Mitotic figures were detectable but were exceedingly rare.

The tumor cells displayed an alternation of cellular and reticular growth patterns. In the former, zellballen-like cellular clusters of neoplastic cells enclosed discrete and sparse vessels. In the latter, trabeculae of neoplastic cells were traversed by abundant slit-like sinusoids. In regions with extensive stromal fibrosis, the tumor cells were dispersed and progressively replaced by hyalinized collagen. The coupled vessels were remained and were frequently dilated (Figure 1F).

**Figure 2:**
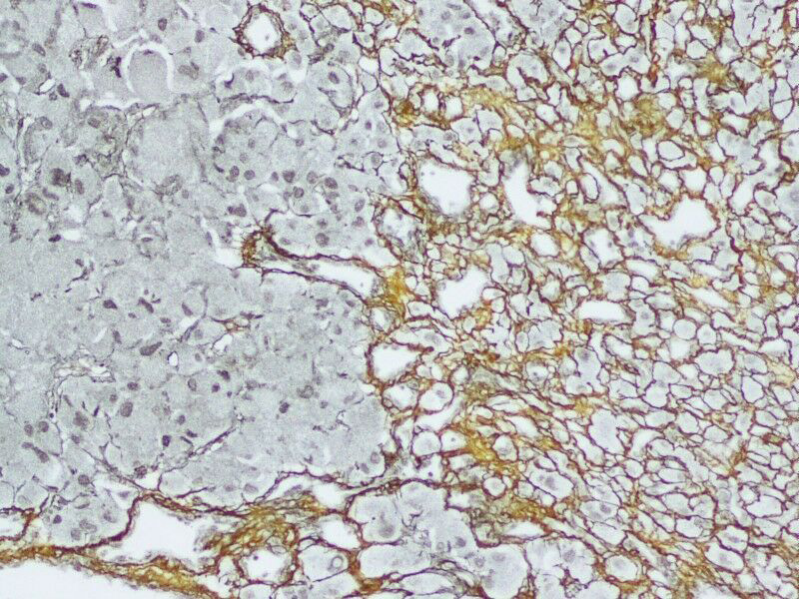
**Reticulins were found around individual tumor cells as well as the vascular channels (reticulin growth pattern, right field)**. Alternatively, only the vascular channels were delineated (cellular growth pattern, left field) (Reticular staining, with original magnification ×200).

Reticular fibers enclosed both tumor cells and vasculature in the areas of reticular growth pattern, but barely surrounded the vessel walls in the regions of cellular growth pattern (Figure 2). PAS staining for glycogen was negative in tumor cells.

**Figure 3:**
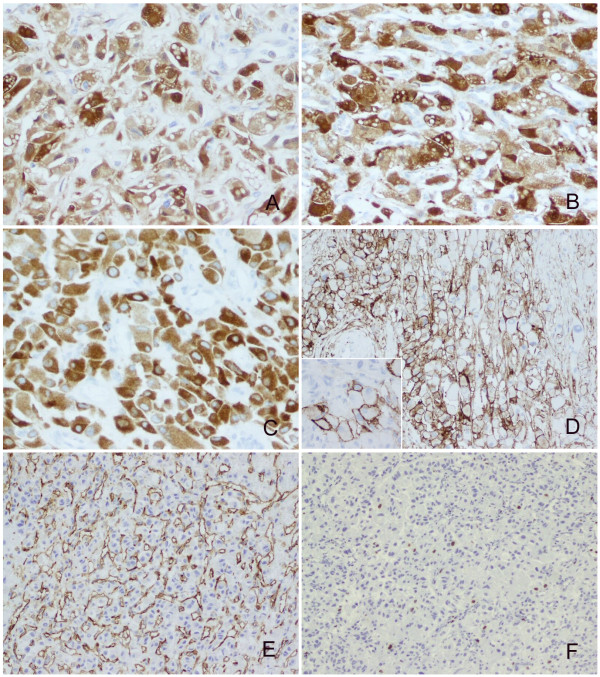
**Immunoprofiles of the renal hemangioblastoma**. The neoplastic cells showed diffuse positive staining for S100 (**A**), NSE (**B**) and a-inhibin (**C**) (with original magnification ×400). Some tumor cells were immunoreactive with CD10 (with original magnification ×200) and bright membrance staining were observed (inserted panel, with original magnification ×400) (**D**). CD34 underlined the rich vascular channels (**E**) (with original magnification ×200). The Ki-67 index was around 1% (**F**) (with original magnification ×100).

The neoplastic cells displayed diffuse immunoreactivity for a-inhibin, NSE, S100, vimentin and EGFR. Focal membranous staining was noted for CD10 and EMA. The Ki-67 index was approximately 1%. CD34 outlined the vascular structures (Figure 3). There was no positive staining for AE1/AE3, CK8/18, CK19, gp200, calretinin, HMB-45, Melan-A, chromogranin, Desmin, Actin, Myoglobin and CD68 (Table 1).

**Table 1:** Panel of immunohistochemical stains

Immunohistochemical stains	Clone	Sources	Dilution	Results
Pan cytokeratin	AE1/AE3	Neomarkers	1:100	Neg.
Keratin 8/18	5D3	Neomarkers	1:200	Neg.
Keratin 19	RCK108	Neomarkers	1:200	Neg.
Renal Cell Carcinoma Marker(gp200)	PN-15	Neomarkers	1:100	Neg.
Neuron-specific enolase (NSE)	NSE-P1	Neomarkers	1:1500	Pos.
Epithelial membrane antigen (EMA)	E29	Neomarkers	1:500	Pos. (focal)
CD10	56 C6	Neomarkers	1:50	Pos. (focal)
Ki-67	SP6	Neomarkers	1:500	Pos. (1%)
Vimentin	SP20	Neomarkers	1:300	Pos.
S100 protein	Polyclonal antiserum	Dako	1:6000	Pos.
a-inhibin	R1	Dako	1:1250	Pos.
Epidermal growth factor receptor (EGFR)	EP38Y	Neomarkers	1:100	Pos.
calretinin	SP13	Neomarkers	1:100	Neg.
Melanoma Marker	HMB45	Neomarkers	1:50	Neg.
Melan-A	A103	Neomarkers	1:100	Neg.
Chromogranin A	SP12	Neomarkers	1:200	Neg.
Desmin	Rabbit Polyclonal Antibody	Neomarkers	1:200	Neg.
Actin	HHF35	Neomarkers	1:100	Neg.
Myoglobin	Rabbit Polyclonal Antibody	Neomarkers	1:800	Neg.
CD68	PG-M1	Neomarkers	1:100	Neg.
CD34	QBEnd/10	Neomarkers	1:80	Neg.

## Discussion

Renal haemangioblastoma (RHB) is extremely uncommon since only four cases were definitely reported previously (Table 2). Our case was specifically in line with the diagnostic clues of RHB suggested by Ip et al [5] and Verine et al [6]. Those characteristics included circumscribed borders, paucity of mitotic figures, fine vacuoles in some tumor cells indicating presence of intracytoplasmic lipids, and a rich capillary network. The immunoprofiles (S100 +, NSE+, a-inhibin + and AE1/AE3-) also conformed to those of haemangioblastomas. However, the majority of the tumor cells in our case showed rhabdoid features, which may be easily mistaken for other rhabdoid tumors that are known to occur in the kidney.

**Table 2:** Clinicopathological characteristics of the reported sporadic hemangioblastomas in the kidney

Case	Age(y)/Sex	Gross features	Histological features				Immunohistochemical staining		Treatment/Follow-up(y)
				
			Cytoplasm	Nuclear	Necrosis	Stromal fibrosis	Diffuse	Focal	
1(ref.4)	71/female	Solid with scattered cystic spaces; located in right kidney	Amphophilic to clear	Round with delicate chromatin; lack of mitoses	Absent	Present	a-inhibin	Vimentin, S100, SMA, MSA, calponin	NP/AW/9
2(ref.5)	58/male	Solid with a cystic cavity; located in right kidney	Eosinophilic	Mildly to highly pleomorphic; lack of mitoses	Absent	Present	a-inhibin, S100, NSE, GLUT1		NP/AW/2
3(ref.5)	55/female	Solid; located in right kidney	Eosinophilic; variable-sized hyaline globules were found	Mildly to highly pleomorphic; very sparse mitoses	Present	Present	a-inhibin, S100, NSE, GLUT1		NP/AW/4
4(ref.6)	64/male	Solid; located in left kidney	Weakly eosinophilic	Enlarged and slightly atypical; lack of mitoses	Absent	Present	S100, NSE, Vimentin	EMA, a-inhibin	pNP/AW/4
5(present case)	61/male	Solid; located in right kidney	Eosinophilic; exhibiting a rhabdoid phenotype	Mildly to highly pleomorphic; intranuclear cytoplasmic pseudoinclusions were present; sparse mitoses	Present	Present	a-inhibin, S100, NSE, EGFR	CD10, EMA	NP/AW/1

NP, nephrectomy; pNP, partial nephrectomy; AW, alive and well

In our opinion, the first differential consideration is the malignant rhabdoid tumors (MRTs). Although most of MRTs afflict young children, there still are sporadic cases affecting adults [7,8]. Several features seen in our case do not support the diagnosis of MRTs: (i) MRTs generally show vesicular chromatin, prominent nucleoli and hyaline intracytoplasmic inclusion [8]. The neoplastic cells in our case demonstrated dark-stained and coarse granular chromatin, and lack the discernable nucleoli as well as cytoplasmic inclusions. (ii) MRTs are devoid of the cytoplasmic lipid droplets and arborizing stromal vasculature, characterized by haemangioblastomas. (iii) Immunohistochemically, MRTs occasionally are focally positive for S100 and NSE [9], but a-inhibin staining was not shown. The features are different from the extensive expression of S100, NSE and a-inhibin seen in RHB. (IV) MRTs are a highly invasive and lethal neoplasm with a proliferation index of Ki-67 around 95% [8]. In contrast, the extremely low Ki-67 index and rare mitosis indicate an indolent behavior of our case.

Another necessary differential consideration is renal cell carcinomas with rhabdoid features (RCCR), which have been previously described [10,11]. RCCR are predominantly composed of large polygonal cells with eccentric nuclei and eosinophilic cytoplasm. Of the 23 cases analyzed by Gökden et al. [10], RCCR showed a diffuse NSE staining (79% of cases) and focal positive staining for EMA (47% of cases) and S-100 (37% of cases), whereas cytokeratin expression was decreased (56% of cases). Obviously, there are many morphologic and immunophenotypic features that markedly overlap with our present case. Nevertheless, compared with RCCR, the tumor cells of our case did not display the vesicular nuclei and prominent nucleoli. Furthermore, RHB is characterized by abundant vascular networks, which are strikingly reduced in RCCR [11]. In addition, RHB demonstrates negative PAS staining for glycogen, whereas this staining is positive in RCCR [11]. The low Ki-67 index in our case is also not compatible with the high proliferative index in RCCR [12].

Other neoplasms with rhabdoid features possibly that need to be considered before making the diagnosis of RHB include epithelioid angiomyolipoma (HMB-45^+ ^and Melan-A^+^), malignant melanoma with rhabdoid features (HMB-45^+ ^and Melan-A^+^) [13], paraganglioma (synaptophysin^+^, chromogranin^+ ^and a-inhibin^-^), epithelioid leiomyosarcoma with rhabdoid features (SMA^+^, desmin^+ ^and S100^-^) [14], and epithelioid malignant peripheral nerve sheath tumor (a-inhibin^-^) [15].

Interestingly, focal CD10 positivity was observed in some tumor cells of our present case. In the normal kidney, CD10 stains glomerular cells and proximal convoluted tubules, and participates in the regulation of water and sodium metabolism [16]. Thus, CD10 expression in RHB seemingly substantiates the earlier hypothesis that haemangioblastomas are derived from pluripotent mesenchymal cells, and partially acquire some site-specific markers of their parental organs during pathogenesis [4]. Many studies have suggested CD10 is a powerful marker in the differentiation between renal cell carcinoma and haemangioblastoma since it usually demonstrates positive staining in renal cell carcinoma while is steadily negative in haemangioblastoma [17]. Our result indicates that caution should be taken on evaluating the differential efficacy of this reagent. Noticeably, Verine et al. [6] reported a negative result of CD10 staining in their case of RHB. The reasons for the discrepancy remain unknown, and probably either reflect the intrinsic disparities of expressional profiles between the two specimens, or may be just caused by different antibodies used.

Some investigators have suggested that RHB would not be as rare if it had got wider recognition as a primary renal tumor [5,6]. So it is undoubtedly necessary to be aware of the clinicopathologic characteristics of RHB. From the available data, RHB commonly occurs in the elderly people (range 55 to 71 years) and there is no sex predilection. The right kidney seems more prone to be affected and the superior pole is likely the preferential site of mass development. Grossly, these tumors displayed a solid cut surface, though cystic changes were occasionally observed. Architecturally, the lesions were consistently composed of cellular and paucicellular regions. In the cellular areas, the tumor could be subclassified as reticular and cellular variants analogous to cerebellar haemangioblastoma [18]. In the paucicellular zones, stromal fibrosis was prominent. Cytologically, the neoplastic cells in RHB generally contained mild to remarkably eosinophilic cytoplasm and frequently outnumbered the clear cells with rich lipid droplets. The nuclei were often enlarged and hyperchromatic, and frequently displayed some pleomorphism. In addition to the positivity for S100, a-inhibin and NSE, focal expression of EMA, SMA, MSA and calponin were also noted [5]. Nevertheless, Cytokeratins, HMB-45, Melan-A, chromogranin, calretinin, and Myoglobin were characteristically negative.

Compared with cerebellar haemangioblastoma (CHB), RHB manifest with some different features. For instance, RHB incidence peaks in the sixth decade compared to the fourth decade of CHB [19]. RHB usually presents as a solid mass, whereas 65 percent of CHB manifest as a cystic mass [20]. The acidophilic cytoplasm and pleomorphic nuclei that are frequently present in renal tumors are usually not seen in CHB. Stromal hyalinization in RHB is also more prominent than that in CHB. However, the immunophenotype and benign behaviors of RHB reflect the essential consistency with CHB.

## Conclusions

We have presented another case of RHB, which demonstrates distinctive rhabdoid features and focal CD10 expression. The tumor cells of RHB generally show abundant acidophilic cytoplasm and pleomorphic nuclei, whereas the characteristic stromal cells with finely-vacuolated lipid droplets are not usually prominent. Diffuse expression of S100, a-inhibin and NSE are very helpful to narrow down the differential diagnoses. The unexpected positive staining of CD10 in RHB should be particularly concerned. RHB have excellent prognosis, and tumor necrosis and nuclear pleomorphism do not seem to affect the prognosis. Most importantly, RHB should be included in the differential diagnosis of primary renal tumors.

## Competing interests

The authors declare that they have no competing interests.

## Authors' contributions

W-HY designed the study, performed the histological and immunohistochemical evaluation, literature review and drafted the manuscript. JL participated in histological diagnosis and immunohistochemical evaluation, literature review and revised the manuscript. JKCC participated in histological diagnosis. All authors read and approved the final manuscript.

## Consent

Written informed consent was obtained from the patient for publication of this case report and accompanying images. A copy of the written consent is available for review by the Editor-in Chief of this Journal.
